# Bacteriophage Transcytosis Provides a Mechanism To Cross Epithelial Cell Layers

**DOI:** 10.1128/mBio.01874-17

**Published:** 2017-11-21

**Authors:** Sophie Nguyen, Kristi Baker, Benjamin S. Padman, Ruzeen Patwa, Rhys A. Dunstan, Thomas A. Weston, Kyle Schlosser, Barbara Bailey, Trevor Lithgow, Michael Lazarou, Antoni Luque, Forest Rohwer, Richard S. Blumberg, Jeremy J. Barr

**Affiliations:** a Department of Biology, San Diego State University, San Diego, California, USA; b Division of Gastroenterology, Hepatology and Endoscopy, Brigham and Women’s Hospital, Harvard Medical School, Boston, Massachusetts, USA; c Department of Biochemistry and Molecular Biology, Biomedicine Discovery Institute, Monash University, Clayton, Victoria, Australia; d School of the Biological Sciences, Monash University, Clayton, Victoria, Australia; e Infection and Immunity Program, Biomedicine Discovery Institute and Department of Microbiology, Monash University, Clayton, Victoria, Australia; f Department of Mathematics and Statistics, San Diego State University, San Diego, California, USA; g Viral Information Institute, San Diego State University, San Diego, California, USA; h Computational Science Research Center, San Diego State University, San Diego, California, USA; Columbia University College of Physicians & Surgeons

**Keywords:** bacteriophages, endocytosis, phage-eukaryotic interaction, symbiosis, transcytosis

## Abstract

Bacterial viruses are among the most numerous biological entities within the human body. These viruses are found within regions of the body that have conventionally been considered sterile, including the blood, lymph, and organs. However, the primary mechanism that bacterial viruses use to bypass epithelial cell layers and access the body remains unknown. Here, we used *in vitro* studies to demonstrate the rapid and directional transcytosis of diverse bacteriophages across confluent cell layers originating from the gut, lung, liver, kidney, and brain. Bacteriophage transcytosis across cell layers had a significant preferential directionality for apical-to-basolateral transport, with approximately 0.1% of total bacteriophages applied being transcytosed over a 2-h period. Bacteriophages were capable of crossing the epithelial cell layer within 10 min with transport not significantly affected by the presence of bacterial endotoxins. Microscopy and cellular assays revealed that bacteriophages accessed both the vesicular and cytosolic compartments of the eukaryotic cell, with phage transcytosis suggested to traffic through the Golgi apparatus via the endomembrane system. Extrapolating from these results, we estimated that 31 billion bacteriophage particles are transcytosed across the epithelial cell layers of the gut into the average human body each day. The transcytosis of bacteriophages is a natural and ubiquitous process that provides a mechanistic explanation for the occurrence of phages within the body.

## IMPORTANCE

Bacteriophages (phages) are viruses that infect bacteria. They cannot infect eukaryotic cells but can penetrate epithelial cell layers and spread throughout sterile regions of our bodies, including the blood, lymph, organs, and even the brain. Yet how phages cross these eukaryotic cell layers and gain access to the body remains unknown. In this work, epithelial cells were observed to take up and transport phages across the cell, releasing active phages on the opposite cell surface. Based on these results, we posit that the human body is continually absorbing phages from the gut and transporting them throughout the cell structure and subsequently the body. These results reveal that phages interact directly with the cells and organs of our bodies, likely contributing to human health and immunity.

## INTRODUCTION

Bacteriophages are viruses that infect bacteria and are the most abundant life form on the planet (1). Phages—short for bacteriophages—are found in almost any environment surrounding us—including the food that we eat—and are responsible for the majority of global genetic diversity. Phages constitute integral components of our gut microbiome, carry a rich repertoire of genes, and impart strong selective pressures on their bacterial hosts (2–4). Our bodies are frequently and continuously exposed to high numbers of phages, and we secrete more than several billion phages per gram of feces (2).

Phages cannot infect eukaryotic cells; the cell surface receptors and intracellular machinery differ too much from their bacterial hosts. Nevertheless, phages freely and profusely penetrate our bodies and the bodies of other higher vertebrates (5–7). They have been detected in the blood and serum of both symptomatic and asymptomatic humans (7–13). Dosing phages to mice via oral feeding and gastric lavage results in an irregular but rapid and repeatable migration of phages into the bloodstream (14, 15). This is followed by the permeation of phages into all major organs of the body, including lung, liver, kidney, spleen, urinary tract, and even the brain—indicating the capacity of these viruses to cross the blood-brain barrier (14–19).

The gut is the largest reservoir of phages in humans (20, 21), and there is evidence that phages utilize various mechanisms to access the body from there. The most rudimentary route proposed is the “leaky gut,” where cellular damage and punctured vasculature at sites of inflammation allow phages to bypass confluent epithelial layers (22, 23). Other proposed mechanisms include “Trojan horse,” whereby a phage-infected bacterium enters or is engulfed by epithelial cells (24–26); “phage display,” which requires homing ligands to be engineered onto viral capsids to mediate cellular recognition and receptor-mediated endocytosis (27–31); and the “free uptake” of phage particles by eukaryotic cells via endocytosis (25, 32–34). There is supporting and contrasting evidence for all of these mechanisms, suggesting that phages may access the body via diverse routes (35). Few attempts, however, have been made to investigate whether phage transcytosis occurs naturally, and consequently, the principal route that phages use to cross confluent epithelial cell layers and subsequently access the body has yet to be identified.

Here, we report a generalized mechanism whereby phages are transported into and across confluent epithelial cell layers. *In vitro* studies demonstrate the rapid, directional transport of diverse phages across cell lines originating from the gut, lung, liver, kidney, and brain. Phage transcytosis across confluent cell layers had a significant preferential directionality for apical-to-basal transport. Correlative light electron microscopy (CLEM) and cell fractionations revealed that phage particles were capable of accessing endomembrane compartments of the eukaryotic cell. Chemical inhibitors suggest that phages transit through the Golgi apparatus before being exocytosed. Approximately 0.1% of total phages applied were functionally transcytosed across the cell layers, with some residual phages remaining within the cell. Based on these results, we estimate that the average adult human body transcytoses approximately 31 billion phages from the gut into the body every day.

## RESULTS

### T4 phage transcytosis across polarized eukaryotic epithelial cells.

**FIG 1:**
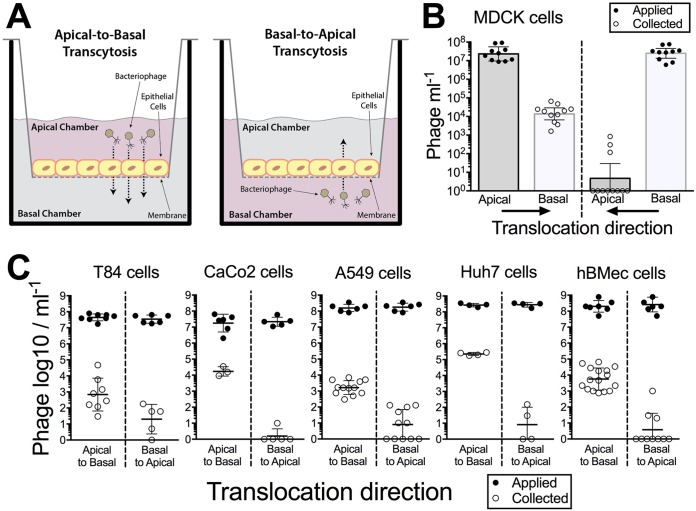
Transcytosis of bacteriophages occurs in a preferential apical-to-basal direction across diverse cell layers. (A) Experimental system to investigate phage transcytosis. Phage T4 was applied to either the apical or basolateral (basal) cell chambers and incubated for 2 h at 37°C, and transcytosed phages were sampled and quantified in the contralateral chamber by bacterial plating. (B) Transcytosis of T4 phages across Madin-Darby canine kidney (MDCK) cells in either an apical-to-basal or basal-to-apical direction. (C) Transcytosis of T4 phages across T84 cells (colon epithelial), CaCo2 cells (colon epithelial), A549 cells (lung epithelial), Huh7 cells (hepatocyte epithelial cell-like), and hBMec cells (brain endothelial). Scatter plots show medians; error bars represent 95% confidence intervals; each point represents the average from three technical replicates.

The directional transcytosis of T4 phage particles across eukaryotic cells was measured using Transwell inserts seeded with Madin-Darby canine kidney (MDCK) cells that were grown to confluence (Fig. 1A). All cells were cultured as high-resistance monolayers to ensure transcytosis across the cell layer, rather than paracellular transport. Average transepithelial resistance (TER) measures were between 150 and 200 Ω ⋅ cm^2^, and postassay confluence was confirmed using Evans blue dye, with all samples falling within the undetectable limits (see Fig. S1 in the supplemental material). Phages were applied to either the apical or the basolateral (basal) side of the cell layer at a mean concentration of 3.2 × 10^7^ phages ml^−1^, and functionally translocated phages were collected and quantified in the contralateral chamber 2 h later by plating with their bacterial host. Apical-to-basal transcytosis ranged from 3.6 × 10^3^ to 6.6 × 10^4^ phages ml^−1^ (Fig. 1B) ([2.0 ± 1.9] × 10^4^, median ± standard deviation [SD]; *n* = 10; coefficient of variation [CV] = 83%), and basal-to-apical transcytosis ranged from 0 to 8.3 × 10^2^ phages ml^−1^ (125 ± 267, median ± SD; *n* = 10; CV = 212%), representing 0.1% and 0.0008% of phages being functionally transcytosed, respectively (Table S1). Phage transcytosis across confluent cell layers had a significant preferential directionality for apical-to-basal transport (Fig. 1B) (Mann-Whitney, *n*_1_ *= n*_2_ = 10, *U* = 0, *P* < 0.0001, two-tailed).

10.1128/mBio.01874-17.2 FIG S1  Postassay confluence test with Evans blue dye. (A) Absorbance of Evans blue standard curve. (B) Zoom-in of absorbance of Evans blue standard curve with mean absorbance of medium control and limit of detection for the assay reported. (C) Postassay Evans blue dye absorbance that was applied to apical wells. (D) Postassay Evans blue dye absorbance collected from basal wells, with mean absorbance of medium control and limit of detection for the assay reported. Absorbance values for all basal samples reported in this work fall within the undetectable limit of Evans blue dye. Download FIG S1, JPG file, 0.4 MB. © Crown copyright 2017. 2017 Crown This content is distributed under the terms of the Creative Commons Attribution 4.0 International license.

10.1128/mBio.01874-17.4 TABLE S1  Directional transcytosis of T4 phages across confluent MDCK monolayers. Download TABLE S1, PDF file, 0.04 MB. © Crown copyright 2017. 2017 Crown This content is distributed under the terms of the Creative Commons Attribution 4.0 International license.

To determine whether the T4 phage transcytosis observed within the MDCK cell line was evidence of a more generalized transport phenomenon, we examined phage transcytosis across cell lines derived from distinct organs and which form confluent monolayers, including those from the gut (T84 and CaCo2), lung (A549), liver (Huh7), and brain (hBMec) (Fig. 1C). All cell types displayed bidirectional transcytosis with a significant preference for the apical-to-basal directionality (Table S2). The transcytosis of T4 phages across these cells indicates a general transport mechanism of phages across polarized epithelial and endothelial cell monolayers with strong apical-to-basal transport directionality.

10.1128/mBio.01874-17.5 TABLE S2  Collected transcytosis of T4 phages across confluent epithelial monolayers. Download TABLE S2, PDF file, 0.1 MB. © Crown copyright 2017. 2017 Crown This content is distributed under the terms of the Creative Commons Attribution 4.0 International license.

### Functionality of phage transcytosis.

**FIG 2:**
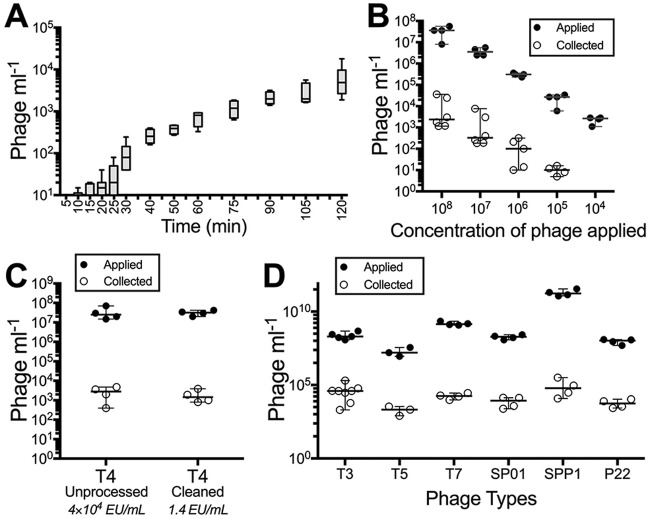
Rate of apical-to-basal phage transcytosis across diverse phages. (A) Rate of T4 phage transcytosis across MDCK cells over a 2 h period. (B) Transcytosis of T4 phage applied to MDCK cells at sequentially decreasing log_10_ concentrations. (C) Transcytosis of unprocessed (4 × 10^4^ endotoxin units [EU] ml^−1^) and cleaned (1.4 EU ml^−1^) T4 phages across MDCK cells. (D) Transcytosis of diverse phage types (T3, T5, T7, SP01, SPP1, and P22 phages) across MDCK cells. Bar plot shows mean; error bars show minimum and maximum values. Scatter plots show medians; error bars represent 95% confidence intervals; each point represents the average from two technical replicates.

The ingress of phages throughout the body has been previously observed (5–11, 14, 15). However, there have been no quantitative measurements of the rate, dose, or generality of the phenomenon. Using T4 phages and MDCK cells, the rate of apical-to-basal transcytosis was recorded over a 2 h period (Fig. 2A). Phages were detected within the basal chamber as early as 10 min after application, with consistent transport occurring within 30 min and steadily increasing up to 2 h. The rate of phage transcytosis was calculated as 0.325 × 10^−12^ ml/(μm^2^ ⋅ h), by per-unit time (hour), surface area (square micrometer), and applied concentration (phages per milliliter) (Text S1). Apical-to-basal transcytosis was found to be dose dependent (Fig. 2B). As the dose of apically applied phage was sequentially reduced by 10-fold, the basal collection sequentially decreased in a proportional manner, continuing down to a dosage of 10^4^ phages ml^−1^, which represented the limit of detection for the assay.

10.1128/mBio.01874-17.1 TEXT S1  Transcytosis and leaky-gut models and equations. Download TEXT S1, DOCX file, 0.1 MB. © Crown copyright 2017. 2017 Crown This content is distributed under the terms of the Creative Commons Attribution 4.0 International license.

Phage preparations are often contaminated by host bacterial macromolecules, with the major pyrogen being lipopolysaccharide (endotoxin) (36). Endotoxin is known to elicit a wide range of pathophysiological effects in the body, stimulating cellular and immune responses (37). To investigate whether residual endotoxins were triggering phage transcytosis, we compared T4 phages before and after removal of endotoxins (38). The removal of endotoxins produced no significant change in apical-to-basal transcytosis of T4 phage (Fig. 2C) (Mann-Whitney two-tailed, *n*_1_ *= n*_2_ = 4, *U* = 6, *P* = 0.6857).

The generality of phage transcytosis was next tested using diverse phages across the order of *Caudovirales*, encompassing phages from three major morphotypes (*Myoviridae*, *Siphoviridae*, and *Podoviridae*) and Gram-positive and -negative bacterial hosts, phages originating from soil and intestinal reservoirs, and different particle sizes. All phages tested elicited apical-to-basal transcytosis (Fig. 2D), although the percentage of phages being functionally transcytosed varied (Table S3).

10.1128/mBio.01874-17.6 TABLE S3  Transcytosis of diverse phages across confluent MDCK epithelial monolayers. Download TABLE S3, PDF file, 0.1 MB. © Crown copyright 2017. 2017 Crown This content is distributed under the terms of the Creative Commons Attribution 4.0 International license.

### Microscopy of phage transcytosis.

**FIG 3:**
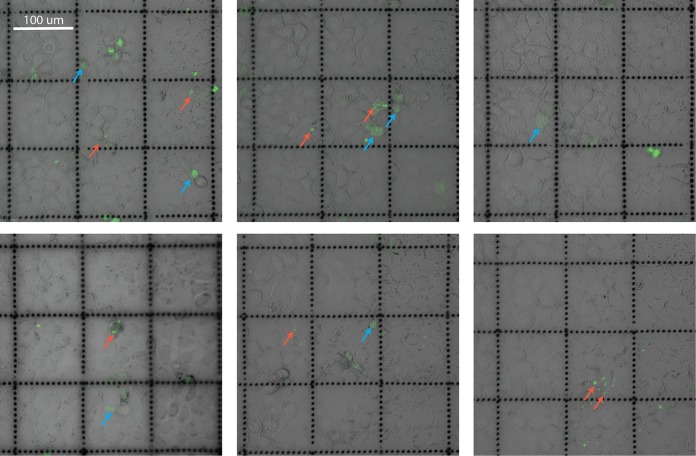
MDCK cells were grown on gridded dishes, incubated with SYBR gold-labeled T4 phages (green), and imaged under differential interference contrast and confocal microscopy. Red arrows indicate cells containing discrete fluorescent signal; blue arrows indicate cells with diffuse fluorescent signal spread across the cytoplasm.

T4 phages were fluorescently labeled using SYBR gold followed by extensive washes to remove excess stain and incubated with MDCK cells for 2 h to allow for uptake. The proportion of fluorescence-positive cells treated with labeled T4 phages was 10.54% (*n* = 2,961; 10.54% ± 0.81%, mean ± standard error [SE]) and 1.7% (*n* = 1,650; 1.7% ± 0.5%, mean ± SE) as analyzed by epifluorescence microscopy and flow cytometry, respectively. These differences in quantification are likely due to a combination of reduced detection of intracellular fluorescence via flow cytometry and the detection of additional cell surface-associated fluorescence signal via microscopy. Next, fluorescently labeled phages were incubated with MDCK cells for 2 h and imaged using confocal microscopy (Fig. 3). Fluorescent signals were visualized as both discrete puncta and diffuse fluorescent clouds spread across the cytoplasm, suggesting that phage particles were present within membrane-bound vesicles and free-floating within the cytoplasm.

**FIG 4:**
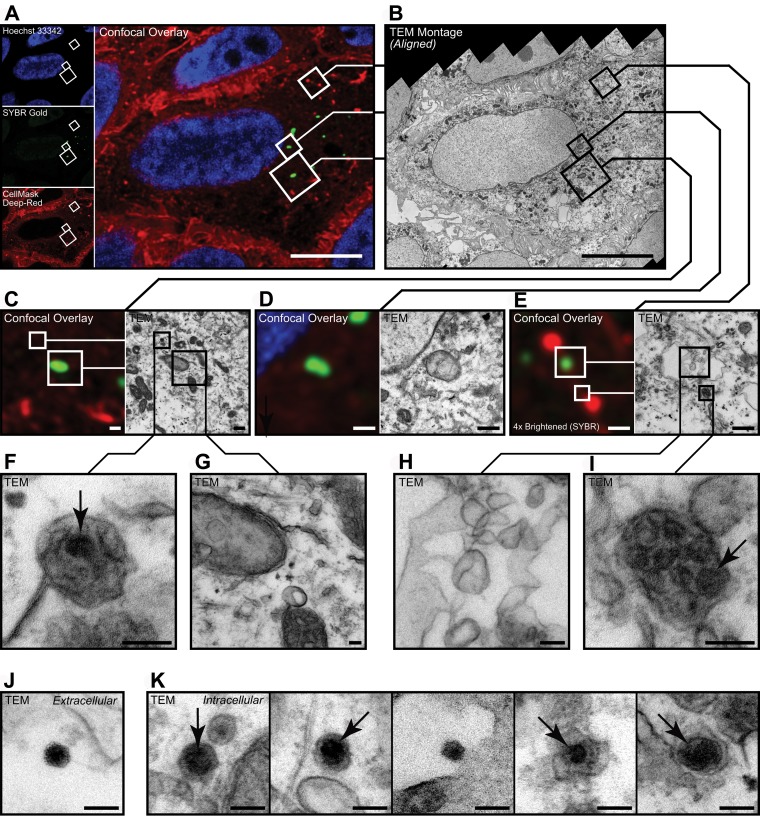
Visualization of intracellular phages. (A to I) Representative correlative micrographs of an MDCK cell stained with Hoechst stain (blue) and CellMask (red) after application of T4 phages stained with SYBR gold (green). (A and B) SYBR gold-positive target cell was selected during confocal microscopy (A) and then processed and aligned for inspection of the same structures by transmission electron microscopy (TEM) (B). (C to I) Spatially aligned electron micrographs showing SYBR gold-positive endomembrane structures (G and H), adjacent to SYBR gold-negative virus particles (F and I). (J and K) Representative electron micrographs of extracellular (J) and intracellular (K) virus particles found in CLEM samples. Data in panel A are maximum projection between the 37th and 43rd optical sections (3.0 µm to 4.2 µm above coverslip surface). Data in panel B are a distortion-corrected TEM montage from the 47th resin section (3,670-nm sample depth, 85 nm thick) acquired at 25 kx. Arrows indicate virus-like particles within membrane-bound vesicles. Data used for spatial alignment are shown in Fig. S2. Bars, 10 µm (A and B), 500 nm (C to E), and 100 nm (F to K).

Next, we selected a SYBR gold-positive target cell using confocal microscopy (Fig. 4A) and processed the cell for ultrastructure inspection of intracellular phage particles using correlative light electron microscopy (CLEM) (Fig. 4B) (39). Membrane-bound virus-like particles were visible within the target cell using transmission electron microscopy (TEM); however, these particles did not colocalize with SYBR gold fluorescence, and conversely, fluorescent signal was found in vesicles that did not appear to contain T4 phages (Fig. 4C to I). The lack of colocalization between SYBR gold fluorescence and virus-like particle ultrastructure may be attributed to numerous factors, including but not limited to: insufficient fluorescent labeling of T4 phages, limited fluorescence detection of individual labeled phages, pH instability of SYBR gold stain (pH 7 to 8.5) under the acidic conditions found within the endosomal lumen (pH 5 to 6.2) (40), or the degradation of labeled phages within the cell. Despite the lack of colocalization, both extracellular and intracellular virus-like particles were found within the SYBR-positive target cell using TEM (Fig. 4F, I, J, and K). Internalized virus-like particles were visualized as electron-dense, icosahedral structures of less than 100 nm, within membrane-bound compartments, suggesting that phages are transcytosed via the endomembrane system.

10.1128/mBio.01874-17.3 FIG S2  Source data used for spatial alignment between optical and electron microscopy. (A) Montage of four-slice grouped maximum projections from the three-dimensional optical data after deconvolution, used to verify target depth for ultramicrotomy. (B) Distortion-corrected TEM montage from the 47th resin section acquired at 25 kx, used for final spatial alignment. Bars, 10 µm. Download FIG S2, JPG file, 9.6 MB. © Crown copyright 2017. 2017 Crown This content is distributed under the terms of the Creative Commons Attribution 4.0 International license.

**FIG 5:**
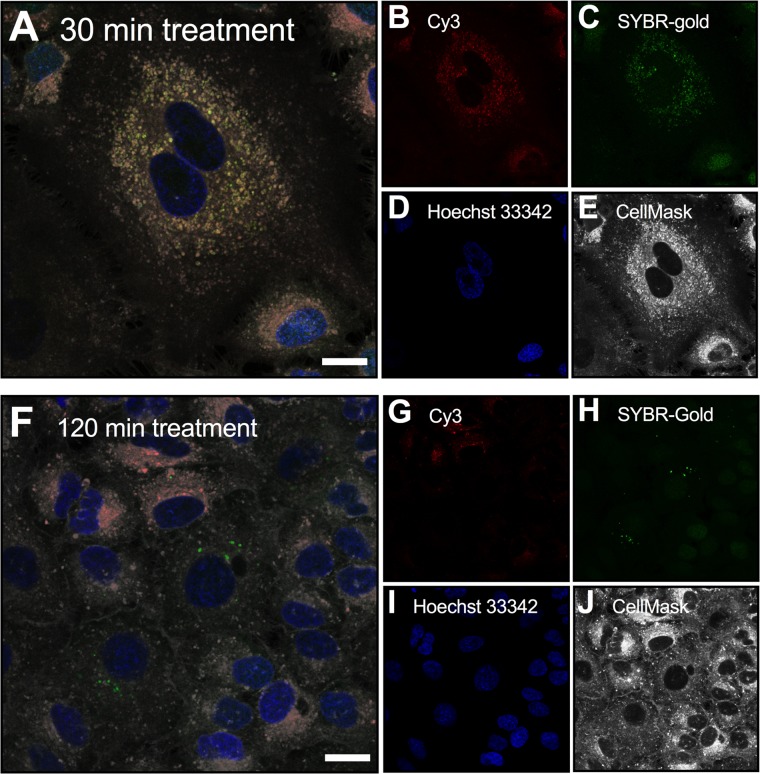
MDCK cells were treated with T4 phage labeled with both the DNA-complexing SYBR gold stain (green) and capsid-conjugated Cy3 stain (red) for either 30 min (A) or 2 h (F) and imaged under confocal microscopy. Cells were stained with Hoechst stain (blue) and CellMask (white) after application of T4 phage. The 30-min treatment sample showed correlation of DNA and capsid fluorescence signals, compared with the 2 h treatment, where there was a disassociation of fluorescence. Bar, 10 μm.

To address the lack of fluorescence and TEM ultrastructure correlation, we performed a time-series experiment using dual-fluorescence-labeled phages that were incubated with MDCK cells for either 30 min or 2 h (Fig. 5). Phages were labeled with both the DNA-complexing SYBR gold stain and a capsid-linked Cy3 stain, followed by imaging using confocal microscopy. Cellular incubations at 30 min revealed correlations between SYBR gold and Cy3 fluorescence within membrane-bound vesicles of the cell (Fig. 5A, B, C, and E). Comparatively, cellular incubations for 2 h (same incubation time used for the CLEM experiment) showed evidence of disassociation of dual-fluorescence signals within the cell, with distinct SYBR gold- and Cy3-positive vesicles observed (Fig. 5F to H). This disassociation of dual-label fluorescence signals suggests that either an instability of fluorescence dyes or the degradation of phage-internalized phage particles occurred and may explain the lack of signal colocalization in the previous CLEM results (Fig. 4). Further work using real-time microscopy approaches, labeling of additional endomembrane compartments, and repeat CLEM analyses using dual-fluorescence-labeled phages is required to further elucidate how phage transcytosis occurs.

### Permeation and inhibition of phages throughout the eukaryotic cell.

**FIG 6:**
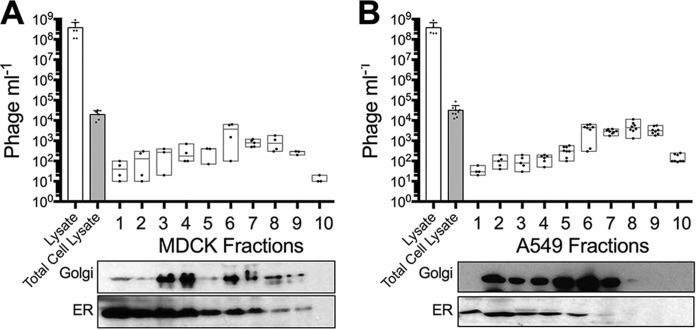
Subcellular fractionation of phage-treated MDCK and A549 cells. (A) Fractionation of MDCK cells treated with T4 phages (lysate) for 18 h. Cells were washed and lysed using the lysosome enrichment kit, and the total number of phages in cells (Total Cell Lysate) was determined. Total cell lysates were fractionated, and 10 cellular fractions were collected and split for either phage quantification by bacterial plating or Western blot analysis of Golgi apparatus and endoplasmic reticulum (ER). (B) Fractionation of A549 cells treated with T4 phages for 18 h. Scatter plots show medians; error bars represent 95% confidence intervals. Bar plot shows mean; error bars show standard deviations.

Next, subcellular fractionation was performed to assess intracellular T4 phage dispersal within MDCK and A549 cells. To ensure maximal uptake and penetration of phages throughout the subcellular structure, cells were incubated with phages for 18 h, extensively washed, fractionated, and the vesicular and cytoplasmic cellular components were collected. Vesicular fractions were then split, with half of the fraction lysed using chloroform and the total number of phages quantified by plating with their bacterial host, and the remaining fraction was protein precipitated and analyzed by immunoblotting using Golgi apparatus and endoplasmic reticulum markers (Fig. 6). Phages accumulated within the total cell lysate (MDCK cells, 2 × 10^4^ ± 1 × 10^4^ [median ± SD], *n* = 5, CV = 53%; A549 cells, 2.6 × 10^4^ ± 2.3 × 10^4^ [median ± SD], *n* = 9, CV = 70%) and were detected in all subcellular fractions of the cell (Table S4). Intracellular phages were found to be enriched within the denser endomembrane fractions of the cell that were associated with the Golgi apparatus.

10.1128/mBio.01874-17.7 TABLE S4  Subcellular fractionation of MDCK and A549 cells treated with T4 phage for 18. Download TABLE S4, PDF file, 0.1 MB. © Crown copyright 2017. 2017 Crown This content is distributed under the terms of the Creative Commons Attribution 4.0 International license.

**FIG 7:**
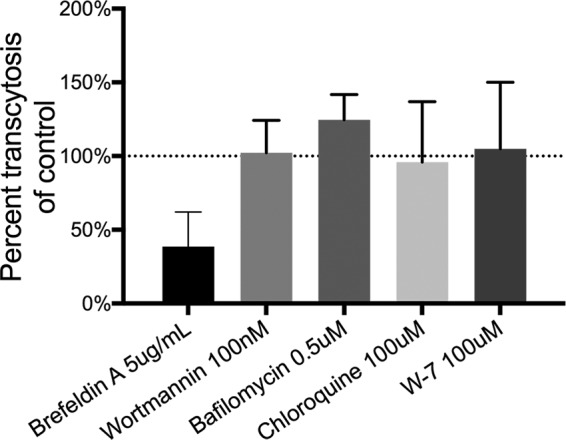
Inhibition of phage transcytosis. Percent transcytosis of T4 phages across MDCK cells pretreated with chemical inhibitors for 18 h compared to a solvent control. Bar plot shows mean; error bars show standard deviations.

The mechanism of phage transcytosis across eukaryotic cells remains ambiguous (25, 27, 32). We applied chemical inhibitors known to arrest steps along the transcytotic pathway to MDCK cells 18 h prior to application of T4 phages. Inhibition of phage transcytosis was reported as the percentage of phages transcytosed across inhibitor-treated cells compared to cells treated with a solvent control (Fig. 7; Table S5). Treatment with brefeldin A, which inhibits post-Golgi-membrane traffic and protein translocation between the endoplasmic reticulum and the Golgi apparatus (41, 42), showed significant but incomplete inhibition of transcytosis, with 38.5% of phages transcytosed compared to a solvent control. We observed no significant treatment effects of wortmannin (an inhibitor of phostadidylinositol-3-kinase and receptor-mediated transcytosis), bafilomycin (an inhibitor of endosomal acidification), chloroquine (an inhibitor of endosomal acidification), or W-7 (an antagonist of calmodulin that inhibits microtubule endocytic membrane transport). This suggests that phages may transit through the Golgi apparatus before being exocytosed via the basolateral membrane (32). However, these inhibitors can impact cellular trafficking in a range of ways, and further direct evidence is needed to confirm this.

10.1128/mBio.01874-17.8 TABLE S5  Inhibition of T4 phage transcytosis across confluent MDCK monolayers by chemical inhibitors. Download TABLE S5, PDF file, 0.1 MB. © Crown copyright 2017. 2017 Crown This content is distributed under the terms of the Creative Commons Attribution 4.0 International license.

### Estimates of phage ingress to the human body.

Within the human body, the largest aggregation of phages resides within the gut (20, 43). Although the concentration of bacteria within the human gut (averaging 9.17 × 10^10^ per g of feces) has been well documented (44, 45), direct quantification of phages is comparatively lacking. Based on three literature references utilizing direct counts and DNA yield, we estimate 5.09 × 10^9^ phage particles per gram of feces (2, 46, 47), yielding 2.09 × 10^12^ phage particles within the colon of an average human (45). Using our experimentally derived rate of phage transcytosis across T84 gut epithelial cells and assuming a 4.4-fold-increased concentration of phages on mucosal surfaces (48), we estimate that the average human body transcytoses 3.1 × 10^10^ phages per day (Text S1). Finally, we contrast this estimate with a competing mechanism of access to the body via a leaky gut. In this model, free phages are allowed to bypass confluent epithelial layers at sites of damage and inflammation, gaining access to the body directly (22, 23). To achieve phage ingress similar to that in our transcytosis model, we estimate that this requires lesions of approximately 256 mm^2^ within the gastrointestinal tract or the removal of 10.24 × 10^6^ epithelial cells (Text S1). This amount of intestinal damage would likely result in significant inflammation of the gut and is in contrast to the detection of phages within the blood and serum of asymptomatic humans (7–13).

## DISCUSSION

The transcytosis of bacteriophages across confluent epithelial cell layers (Fig. 1) provides a mechanistic explanation for the occurrence of phages within the human body in the absence of disease (8–10, 12, 13) and supports the establishment of the intrabody phageome (35). Apical-to-basal transcytosis was observed with every phage type investigated across diverse cell lines (Fig. 1C and 2D), with paracellular transport across an intact epithelial barrier not likely to be a significant mechanism. Microscopy investigations combining fluorescence and TEM revealed phages present within both membrane-bound vesicles (Fig. 3 and 4F, I, and K) and provided evidence that phages were capable of accessing the cytosol (Fig. 3). The purpose of the CLEM experiment was to detect phage particles within EM images of the cell guided by a fluorescence signal. However, virus-like particles in EM images did not correlate with confocal fluorescence signal, and conversely, fluorescence signal was found in vesicles that did not contain virus-like particles. To address this inconsistency, we imaged dual-fluorescence-labeled phages and revealed a loss of fluorescence signal correlation within endomembrane compartments of the cell over time (Fig. 5). From these experiments, we conclude that SYBR gold-labeled phages are endocytosed by epithelial cells and that this signal can be used to positively identify cells that have endocytosed phages. However, either SYBR gold fluorescence was unstable on internalized phage particles or the phage particles were degraded and recycled within the endomembrane system over time, and we attribute these limitations to the lack of signal correlation between virus-like particle fluorescence and EM ultrastructure in the CLEM experiment. Cellular investigations showed that phages were capable of accessing all subcellular fractions of the eukaryotic cell (Fig. 6) with intracellular transport suggested to traffic through the Golgi apparatus (Fig. 7). The strong apical-to-basal transport suggests that epithelial cells are preferentially transporting phages in this direction. Based on these results, we estimate that 31 billion phage transcytotic events occur within the average human body per day, while comparable ingress via leaky gut is estimated to require significant damage and inflammation to the gastrointestinal tract (22, 23).

If these epithelial cell layers and, by extension, the human body are perpetually absorbing phages, what might be their intended function? The major reservoir of phages within the body is observed in the gastrointestinal tract. These gut phages coevolved with the microbiome over the course of our life span, engage in tripartite symbioses, and represent the most genetic diversity and “biological dark matter” in the body (48–50). At the simplest level, the presence of a low-level but continuous stream of phages originating from the gut and disseminating through the blood, lymph, and organs may provide the host with a system-wide antimicrobial against the intrusion of any opportunistic gut microbe (6, 51). Their dissemination may have additional roles in cellular disease, cancer recognition, and even the vertical transmission of adapted gut phage populations from mother to infant through breast milk (52–54).

Conversely, phages are foreign and immunogenic particles capable of stimulating humoral immune responses and inducing antiphage antibodies (55, 56). How then can our body sustain a persistent influx of immunogenic particles without eliciting inflammatory immune responses? Transcytosed gut phages are likely continuously dosed to the body at relatively low levels, lack costimulatory factors such as endotoxins, and reflect the high diversity of the gut microbiome. As such, the immunostimulatory effects of transcytosed phages on the body are largely unknown. Nonetheless, their presence within the body could provide long-term immunologic tolerance through interactions with regulatory T cell populations and downregulation of specific and nonspecific immune reactions and contribute to immune homeostasis in the gut (6, 57). Alternatively, aberrant transcytosis may contribute to enhanced immune responses, allergic reactions, and inflammatory diseases.

Perhaps the greatest potential function of transcytosed gut phages is the body’s direct utilization of their astounding genetic diversity. Previous work using recombinant phages has already documented the delivery and expression of single or multiple genes to eukaryotic cells both *in vitro* and *in vivo* (28, 58). The transcytosis of diverse phages reported here provides a mechanism to both traverse and disseminate throughout the eukaryotic cell. This intracellular dissemination of phages and their genetic material provides a means to directly affect the eukaryotic cell. Although we did not directly observe phage particles or DNA within the nucleus, prior research on phage gene delivery and the complete permeation of epithelial cells by phages shown here suggest a potential route that requires further investigation (28, 58). Nominally, this allows for horizontal gene transfer between phages and eukaryotes (59) and the transcription and translation of phage genetic material within epithelial cells and the body (58) and potentially represents an unexplored “third external genome” (60). Additional research is required to elucidate the intracellular effects of phages within the eukaryotic cell and the mechanism of phage intracellular transit and release from endocytic vesicles and to characterize direct phage-eukaryote interactions. Our studies suggest that the transcytosis of bacteriophages across confluent epithelial cells is a naturally occurring and ubiquitous process that adds credence to the use and application of phages in a biomedical setting.

## MATERIALS AND METHODS

### Bacterial strains, phage stocks, tissue culture cell lines, and growth conditions.

*Escherichia coli* B strain HER 1024 was grown in LB (10 g tryptone, 5 g yeast extract, 10 g NaCl, in 1 liter distilled water [dH_2_O]) at 37°C with shaking overnight and used to propagate and quantify bacteriophages T4, T3, T5, and T7. *Bacillus subtilis* 168WT was grown in TY broth (10 g tryptone, 5 g yeast extract, 5 g NaCl, 10 mM MgSO_4_, 100 μM MnSO_4_, in 1 liter dH_2_O) at 37°C with shaking for 6 to 8 h and used to propagate and quantify bacteriophages SP01 and SPP1, which were kindly supplied by Ryland Young. *Salmonella enterica* serovar Typhimurium LT2 was grown in LB at 37°C with shaking overnight and used to propagate and quantify bacteriophage P22. All phage lysates were purified and cleaned of bacterial endotoxins according to the Phage-On-Tap protocol (38). All tissue culture cell lines were grown at 37°C and 5% CO_2_ and supplemented with 1% penicillin-streptomycin (Mediatech, Tewksbury, MA). MDCK.2 cells were grown in Eagle’s minimal essential medium with 10% fetal bovine serum (FBS), T84 cells were grown in Ham’s F-12 medium and Dulbecco’s modified Eagle’s medium with 2.5 mM l-glutamine with 10% FBS, CaCo2 cells were grown in Eagle’s minimal essential medium with 10% FBS, A549 and Huh7 cells were grown in F-12K medium with 10% FBS, and hBMec cells were grown in RPMI medium with 10% nuSerum (Corning, NY) and 1% non-essential amino acids (NEAA) (Gibco, Waltham, MA).

### Transwell experimental setup.

Transwell polyethylene terephthalate (PET) 12-well plates with 0.4-μm pore size (Corning, NY) were used for all transcytosis assays. All cells were seeded at a density of 0.5 1 × 10^6^ to 1 × 10^6^ cells per well and allowed to grow to confluence (3 to 5 days). For apical-to-basal transcytosis, the apical wells were incubated in Hanks buffered salt solution (HBSS) at pH 6.0 and basal wells were incubated in HBSS at pH 7.4 for 2 h to mimic pH-dependent uptake (61). For basal-to-apical transcytosis, the buffers were switched. Bacteriophages were applied with the HBSS pH 6.0 buffer and incubated with cells for 2 h, and phages from both apical and basal cell layers were quantified by plating with the bacterial host. Cell layer confluence of all Transwell experiments was measured in three separate ways to ensure phage transcytosis across the cell layer, rather than by paracellular transport. First, there was a visual inspection using a phase-contrast microscope. Second, transepithelial resistances (TERs) of all cell lines were measured (World Precision Instruments, Inc., Sarasota, FL), with the acceptable range of measurements being between 150 and 200 Ω ⋅ cm^2^. TER measurements were taken before and after all transcytosis experiments to ensure that cell confluence and polarization had been reached and maintained. Finally, 250 μl of HBSS buffer with 25 μl of Evans blue dye was added to the apical chambers of all Transwells postassay and incubated with cells at 37°C for 2 h. Basal chambers were collected, and absorbance was measured (620 nm) using a spectrophotometer and was compared against an Evans blue dilution curve (see Fig. S1 in the supplemental material). The presence of dye in the basal chamber was indicative of a nonconfluent cell layer, and data from these wells were discarded.

### Confocal microscopy.

For confocal microscopy experiments, MDCK cells were cultured in complete medium for 1 to 3 days on a μ-Slide 8-well glass-bottom slide (Ibidi, Germany). Phages (1 × 10^8^ phages ml^−1^) were labeled with 100× SYBR gold (Invitrogen, USA) for 2 h in the dark followed by extensive washes in Amicon Ultra 0.5-ml centrifugal filter units and a 50-kDa membrane (Merck Millipore, Germany) with Dulbecco’s phosphate-buffered saline (DPBS) buffer to remove excess stain. Dual-fluorescence-labeled phages were then incubated with Cy3 monoreactive dye (GE Healthcare, USA) in 0.1 M sodium carbonate buffer (pH 9.3) for 1 h in the dark followed by extensive washes in Amicon Ultra 0.5-ml filters with DPBS buffer to remove excess stain. Labeled phages were then applied to MDCK cells in complete medium for 2 h at 37°C and 5% CO_2_. Cells were washed with warm DPBS and stained with 2.5 μg/ml CellMask deep red plasma membrane stain (Thermo Fisher, USA) and 1 µM Hoechst 33342 stain (Thermo Fisher, USA) for 30 min at 37°C and 5% CO_2_. The stained cells were aspirated and then fixed using prewarmed phosphate-buffered 4% paraformaldehyde at 37°C and 5% CO_2_ for 2 h. Cells were washed three times in DPBS and stored until imaging. Cells were imaged via confocal microscopy on a Leica Sp8 HyD inverted confocal microscope, using a 63× objective lens.

### CLEM.

For correlative light electron microscopy (CLEM), MDCK cells were cultured in complete medium for 1 to 3 days in a 35-mm 500-grid plastic-bottom μ-Dish (Ibidi, Germany). Phages (1 × 10^8^ phages ml^−1^) were labeled with 100× SYBR gold (Invitrogen, USA) for 2 h in the dark followed by extensive washes in Amicon Ultra 0.5-ml centrifugal filter units and a 50-kDa membrane (Merck Millipore, Germany) with DPBS buffer to remove excess stain. Labeled phages were then applied to MDCK cells in complete medium for 2 h at 37°C and 5% CO_2_. Cells were washed with warm DPBS and stained with 2.5 μg/ml CellMask deep red plasma membrane stain (Thermo Fisher, USA) and 1 µM Hoechst 33342 stain (Thermo Fisher, USA) for 30 min at 37°C and 5% CO_2_. The stained cells were aspirated and then fixed using prewarmed phosphate-buffered 4% paraformaldehyde at 37°C and 5% CO_2_ for 2 h. The fixed sample was imaged on an inverted Leica SP8 confocal laser scanning microscope equipped with a 40×/1.10 objective (water immersion, HC PLAPO, CS2; Leica Microsystems, Inc.) using an HyD hybrid detector (Leica Biosystems) through the Leica Application Suite X (LASX v2.0.1). The optical data (35-nm lateral voxel resolution; 200-nm axial pixel resolution) were deconvolved for subsequent alignment (fast classic maximum likelihood estimation; signal-to-noise ratio of 20; 20 iterations; 0.05 quality threshold) using Huygens Professional (v15.10; Scientific Volume Imaging). After optical imaging acquisition, the sample was postfixed overnight with 2.5% glutaraldehyde in 0.1 M sodium cacodylate buffer at 4°C, rinsed twice with 0.1 M sodium cacodylate, and then osmicated with ferricyanide-reduced osmium tetroxide {1% [wt/vol] OsO_4_, 1.5% [wt/vol] K_3_[Fe(CN)_6_], 0.065 M cacodylate buffer} for 2 h at 4°C and thoroughly rinsed five times using MilliQ water. All subsequent stages were microwave assisted using a BioWave Pro microwave system (Pelco). The sample was *en bloc* stained with 2% (wt/vol) aqueous uranyl acetate using three microwave duty cycles (120 s on and 120 s off) at 100 W under vacuum and then rinsed five times with MilliQ water. Microwave-assisted dehydration was performed at atmospheric pressure using 150 W for 40 s per stage of a graduated series of ethanol (50%, 70%, 90%, 100%, and 100%) and propylene oxide (100% and 100%), and microwave-assisted resin infiltration was performed under vacuum at 250 W for 180 s per stage using a graduated series of Procure-Araldite (25%, 50%, 75%, 100%, and 100%) in propylene oxide before resin polymerization at 60°C for 48 h. The target depth within the target cell was then relocated within the resin block, using the procedure outlined previously (39, 62). The resin block was trimmed and then sectioned using an Ultracut UCT ultramicrotome (Leica) equipped with a 45° diamond knife (Diatome) to cut serial sections (*n* = 49; average thickness, 79 nm) for collection on nine separate 300-mesh hex thin-bar copper grids. Grids containing the sections closest to the target z-planes (3 to 4 µm above coverslip; final 9 sections, 3,245-nm to 3,925-nm depth) were stained at room temperature using 2% (wt/vol) aqueous uranyl acetate (10 min) and Reynolds lead citrate (3 min). TEM imaging was conducted at 80 kV on a Hitachi H-7500 TEM using a Gatan 791 MultiScan side-mount charge-coupled device (CCD) camera and DigitalMicrograph (version 1.71.38) acquisition software. The target cell was relocated, and a TEM montage of 253 images was manually acquired at ×25,000 magnification. The image montage was corrected for EM lens distortion and stitched together using the appropriate plug-ins (63, 64) in FIJI (FIJI Is Just ImageJ, version 1.51h). Within GIMP (GNU Image Manipulation Program, version 2.8.2), the distortion-corrected TEM montage was aligned with the deconvolved optical data using filopodia, the nucleus, and other intrinsic features as anchor points. All subsequent TEM data were aligned directly to the distortion-corrected TEM montage. Correlated fluorescence data were obtained by scaling and aligning TEM images to the TEM montage, extracting the aligned region from the fluorescence channels, and then performing the reverse operations with bicubic interpolation.

### Subcellular fractionation.

MDCK and A549 cells grown to confluence were incubated with T4 phages (1 × 10^8^ phages ml^−1^) for 18 h. Cell layers were then extensively washed with DPBS and subjected to microsomal fractionation (65), using the lysosomal enrichment kit for tissue and cultured cells according to the manufacturer’s instructions (Thermo Fisher). Briefly, ~90% confluent T1 75-cm^2^ flasks were harvested with trypsin and centrifuged for 2 min at 850 × *g*. Lysosome enrichment reagent A containing a protease inhibitor cocktail (Calbiochem) was added to pelleted cells, mixed by vortex at medium speed for 5 s, and incubated on ice for exactly 2 min. Cells were then lysed by sonication with 15 1-s pulses, followed by addition of lysosome enrichment reagent B containing a protease inhibitor, and mixed by inverting. Cells were then centrifuged for 10 min at 500 × *g* at 4°C. The supernatant was then collected, and the final concentration was altered to 15% with OptiPrep cell separation medium. Cell lysates were then overlaid on top of a discontinuous 17:20:23:27:30% (vol/vol) OptiPrep gradient prepared with 1.9 ml of each fraction in a 13.2-ml ultracentrifugation tube (Beckman-Coulter) and centrifuged in an SW40 Ti rotor at 100,000 × *g* for 19 h at 4°C. After ultracentrifugation, gradients were fractionated into 10 equal fractions. Five hundred microliters of each fraction was taken for phage quantification by plating with the bacterial hosts. The remainder of each fraction was trichloroacetic acid (TCA) precipitated (10% final concentration), separated by 12% SDS-PAGE, and analyzed by immunoblotting against Golgi apparatus (anti-GM130, 1:1,000; Abcam, Inc.) and endoplasmic reticulum (anti-ERp57, 1:1,000; Abcam, Inc.) cellular markers.

### Mathematical modeling.

The number of phages transcytosed per day in humans was extrapolated using ratio relations based on the experiments presented here (see details in Text S1). The flow of phages in the leaky-gut mechanism was calculated using Fick’s law, continuous equation, and the Einstein-Smoluchowski equation (see details in Text S1).

### Graphing and statistics.

Graphing and statistical analyses were performed using GraphPad Prism 7 (GraphPad Software, Inc.). Individual data points, medians, and standard deviations were reported where possible (66). Both nonparametric and parametric statistical analyses were performed, although most data did not pass a normality test.
