# Correction for Nguyen et al., “Bacteriophage Transcytosis Provides a Mechanism To Cross Epithelial Cell Layers”

**DOI:** 10.1128/mBio.02207-17

**Published:** 2018-01-02

**Authors:** Sophie Nguyen, Kristi Baker, Benjamin S. Padman, Ruzeen Patwa, Rhys A. Dunstan, Thomas A. Weston, Kyle Schlosser, Barbara Bailey, Trevor Lithgow, Michael Lazarou, Antoni Luque, Forest Rohwer, Richard S. Blumberg, Jeremy J. Barr

**Affiliations:** a Department of Biology, San Diego State University, San Diego, California, USA; b Division of Gastroenterology, Hepatology and Endoscopy, Brigham and Women’s Hospital, Harvard Medical School, Boston, Massachusetts, USA; c Department of Biochemistry and Molecular Biology, Biomedicine Discovery Institute, Monash University, Clayton, Victoria, Australia; d School of the Biological Sciences, Monash University, Clayton, Victoria, Australia; e Infection and Immunity Program, Biomedicine Discovery Institute and Department of Microbiology, Monash University, Clayton, Victoria, Australia; f Department of Mathematics and Statistics, San Diego State University, San Diego, California, USA; g Viral Information Institute, San Diego State University, San Diego, California, USA; h Computational Science Research Center, San Diego State University, San Diego, California, USA

## AUTHOR CORRECTION

Volume 8, no. 6, e01874-17, 2017, https://doi.org/10.1128/mBio.01874-17. The following funding information and acknowledgments should be added:

This work, including the efforts of Benjamin S. Padman and Michael Lazarou, was funded by an Australian Research Council (ARC) Future Fellowship (FT1601100063) and a National Health and Medical Research Council (NHMRC) grant (GNT1106471).

We acknowledge the Monash Ramaciotti Centre for Cryo Electron Microscopy for the use of facilities.

