# Supplementary figures and images for: Bacteriophage Transcytosis Provides a Mechanism To Cross Epithelial Cell Layers
Source: mBio. 2017 Nov 21;8(6):e01874-17. doi: 10.1128/mBio.01874-17 (PMC5698557; doi:10.1128/mBio.01874-17)

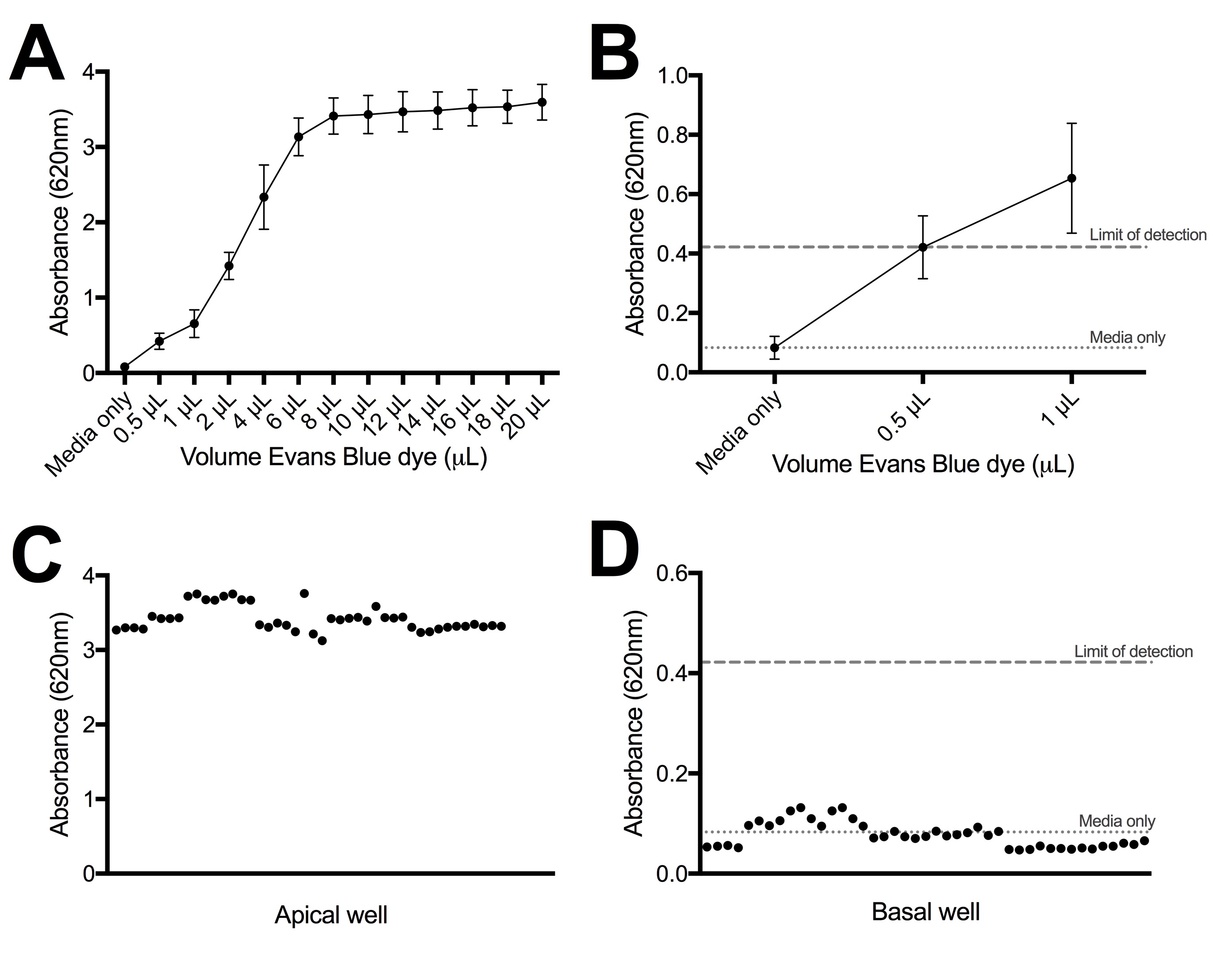

Supplement: FIG S1 [file mbo006173601sf1.jpg]

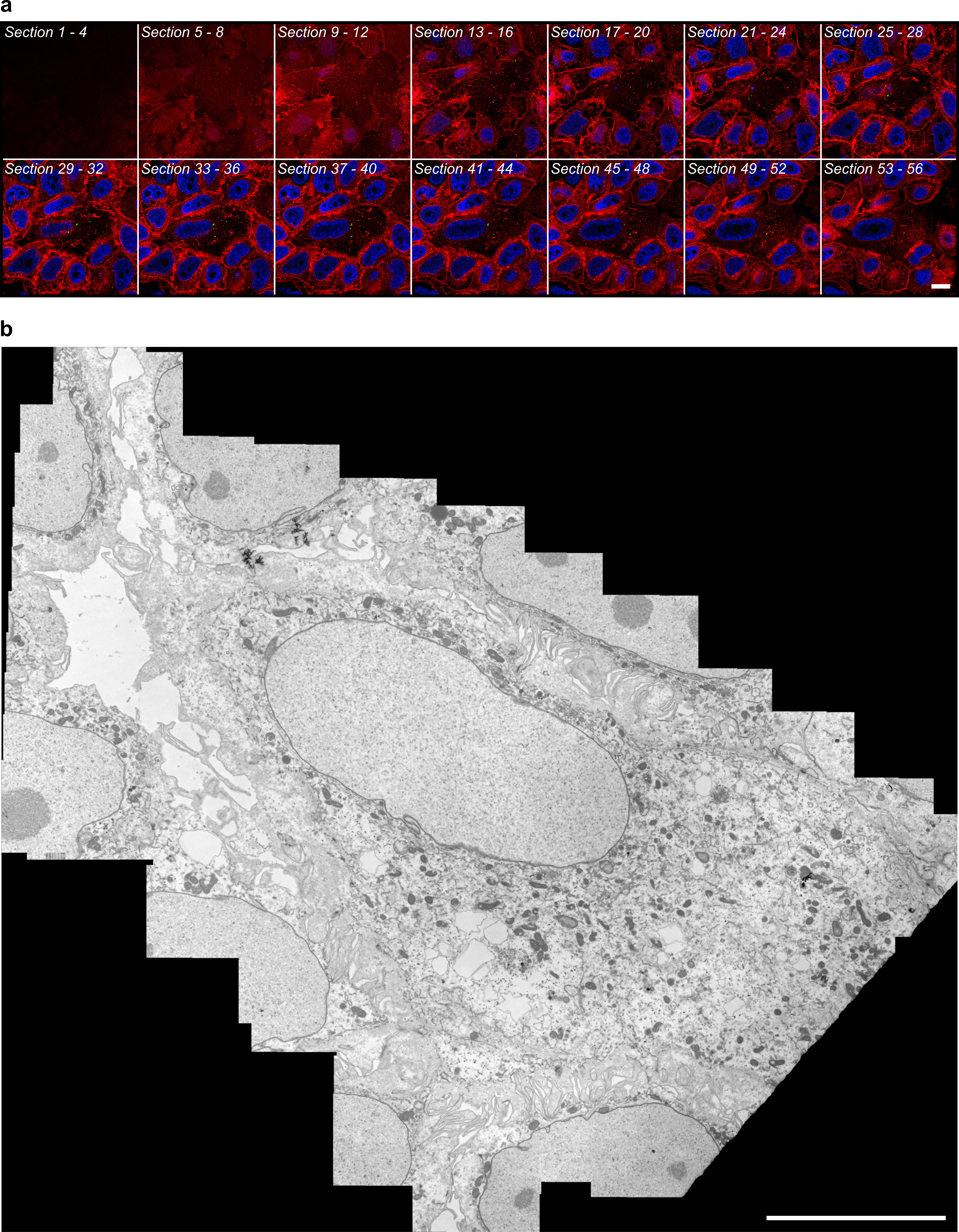

Supplement: FIG S2 [file mbo006173601sf2.jpg]
