# Supplementary material for: Bacteriophage Transcytosis Provides a Mechanism To Cross Epithelial Cell Layers
Source: mBio. 2017 Nov 21;8(6):e01874-17. doi: 10.1128/mBio.01874-17 (PMC5698557; doi:10.1128/mBio.01874-17)
Supplement: TABLE S1 [file mbo006173601st1.pdf]

| Apical-to-basal transcytosis | | | Basal-to-apical transcytosis | | |
|---|---|---|---|---|---|
| **Applied** | **Collected** | **Percentage** | **Applied** | **Collected** | **Percentage** |
| $1.1 \times 10^7$ | $5 \times 10^3$ | 0.05% | $6 \times 10^6$ | $3 \times 10^2$ | 0.005% |
| $1.8 \times 10^7$ | $1.9 \times 10^4$ | 0.1% | $1.2 \times 10^7$ | 0 | 0% |
| $1.2 \times 10^7$ | $3.7 \times 10^3$ | 0.03% | $8 \times 10^6$ | 0 | 0% |
| $2.5 \times 10^7$ | $1.2 \times 10^4$ | 0.05% | $2.8 \times 10^7$ | 0 | 0% |
| $8.7 \times 10^6$ | $1.3 \times 10^4$ | 0.14% | $2.7 \times 10^7$ | $1.2 \times 10^2$ | 0.0004% |
| $3.8 \times 10^7$ | $6.6 \times 10^4$ | 0.17% | $7.6 \times 10^7$ | 0 | 0% |
| $9.2 \times 10^6$ | $2.5 \times 10^4$ | 0.27% | $7.2 \times 10^7$ | 0 | 0% |
| $3.3 \times 10^7$ | $4.8 \times 10^4$ | 0.15% | $3.1 \times 10^7$ | 0 | 0% |
| $9.2 \times 10^7$ | $2 \times 10^4$ | 0.02% | $3.5 \times 10^7$ | 0 | 0% |
| $8.6 \times 10^7$ | $2.5 \times 10^4$ | 0.03% | $3.2. \times 10^7$ | $8.3 \times 10^2$ | 0.0026% |
