# Supplementary material for: Bacteriophage Transcytosis Provides a Mechanism To Cross Epithelial Cell Layers
Source: mBio. 2017 Nov 21;8(6):e01874-17. doi: 10.1128/mBio.01874-17 (PMC5698557; doi:10.1128/mBio.01874-17)
Supplement: TABLE S2 [file mbo006173601st2.pdf]

Cells	Apical-to-basal transcytosis			Basal-to-apical transcytosis			Mann-Whitney two tailed		Paired t-test		
							Non-parametric		*Parametric*		
**Cells**	**Median ± s.d.**	***n***	**CV**	**Median ± s.d.**	***n***	**CV**	**U**	***P***	***t***	***df***	***P***
MDCK	$1.95 \pm 1.94 \times 10^4$	11	90%	1 ± 267	11	224%	0	<0.0001	3.8	9	0.0042
T84	$.79 \pm 1.74 \times 10^4$	7	218%	60 ± 72	5	118%	2	0.008	2.3	3	0.1031
CaCo2	$1.6 \pm 1.3 \times 10^4$	3	67%	1 ± 4	5	143%	0	0.0179	2.6	2	0.1231
A549	$1.6 \pm 2.2 \times 10^3$	12	89%	10 ± 54	11	137%	0	<0.0001	3.6	10	0.0046
Huh7	$2.3 \pm .47 \times 10^5$	4	20%	15 ± 71	4	156%	0	0.0286	3	9	0.0148
hBMec	$.48 \pm 2 \times 10^4$	16	131%	1 ± 314	10	297%	3	<0.0001	9.7	3	0.0024
