# Supplementary material for: Bacteriophage Transcytosis Provides a Mechanism To Cross Epithelial Cell Layers
Source: mBio. 2017 Nov 21;8(6):e01874-17. doi: 10.1128/mBio.01874-17 (PMC5698557; doi:10.1128/mBio.01874-17)
Supplement: TEXT S1 [file mbo006173601s1.docx]

**SUPPLEMENTAL MODEL**

**Phage transcytosis model**

***Phage transcytosis rate.*** We calculate the transcytosis rate per unit time, surface area (wall), and concentration of T4 phages for T84 gut epithelial cells. In a first approximation, this leads to

|  | 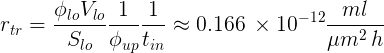 | Eq. (S1) |
| --- | --- | --- |

Here *ϕ_lo_* is the basal concentration of phages, *V_lo_* is the volume of the lower compartment, *S_lo_* is the surface area of a single Transwell, *ϕ_up_* is the apical concentration of phages, and *t_in_* is the time of incubation. The first term (*ϕ_lo_V_lo_/S_lo_*) represents the number of phages that accomplished transcytosis per unit area of the Transwell. This number depends on how many phages contact the epithelial cells on the apical side. Thus, we divide by the apical concentration of phages (*ϕ_up_*). This assumes that the transcytosis mechanism is independent on the number of phages contacting the cell and being transported at a given time. The duration of the experiment will also impact the number of phages that are counted in the basal part of the cell. Thus, we divide by the time of incubation (*t_in_*), which assumes that the rate of the transcytosis mechanism is approximately constant during the time scale of the experiment. Applying the data obtained for the T84 experiments (Table 1). This leads to a transcytosis rate, *r_tr_*, of 0.166 x 10^-12^ ml/(μm^2^h), which is given per unit time, surface area (wall) and apical phage concentration.

***Number of transcytosed phages in humans.*** We estimate the number of phages that are being transcytosed in one day (24 hours) in the average human body. To calculate this number we combine the experimentally derived transcytosis rate with physiological data (Table 2). Using the model in Eq. (S1), the number of phages transcytosed in humans per day is

|  | 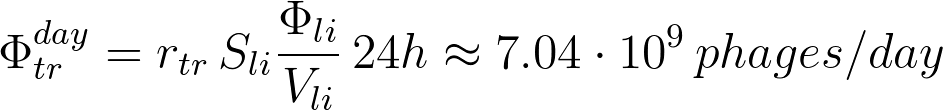 | Eq. (S2) |
| --- | --- | --- |

In this equation, we multiply the transcytosis rate (*r_tr_*) by the surface area of the large intestine (*S_li_)* times the concentration of phages in the intestine (*Φ_li_/V_li_*) times 24 hours (1 day). In this way, we estimate that there are seven billion phages that penetrate the human body per day using the transcytosis route.

***Mucus factor.*** We then assume a 4.4-fold increase of phage numbers associated with mucosal surfaces in the large intestine, giving a total of

|  | 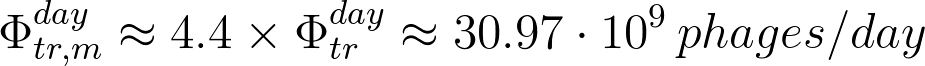 | Eq. (S3) |
| --- | --- | --- |

Thus, there are approximately thirty-one billion phages transcytosed by the human body per day. The constants used in this model are reported in Table 2 (physiological parameters in the large intestine), Table 1 (parameters of the transcytosis experiment), and Table 3 (model results).

***Transcytosis in MDCK cells.*** The results above were based on the experiments done in T84 cells. Here we calculate the factor required to extend the results to Madin–Darby canine kidney cells (MDCK). In T84 cells, when 4.7 × 10^7^ phage ml^-1^ are applied to the apical side and 7.9 × 10^3^ phage ml^-1^ were recovered in the basal side. This leads to a raw transcytosis ratio of 1.48 × 10^-4^. In MDCK cells, 3.2 × 10^7^ phage ml^-1^ were applied to the apical side, and 1.9 × 10^4^ phage ml^-1^ were recovered in the basal side, that is, a raw transcytosis ratio of 2.9 × 10^-4^. Thus, the transcytosis ratio is 1.96 times higher in MDCK cells than in T84 cells. To estimate the transcytosis in MDCK we multiply our modelled results of T84 by ƒ ≈ 1.96, giving a transcytosis rate, *r_tr_*, of 0.325 x 10^-12^ ml/(μm^2^h) per unit time, surface area, and applied phage concentration.

**Phage leaky-gut model**

We assume phages can bypass confluent epithelial layers at sites of inflammation caused by cellular damage and punctured vasculature. Here we introduce a mathematical model to estimate the flux of phages penetrating the body using this route. The constants used in and the values obtained from the model are summarized in Table 4.

***Leaky-gut model upper bound limit.*** In a first approximation, we consider that every damaged region in the gut is equivalent to removing an entire epithelial cell, thus opening a channel 40um long (Table 2), and we assume that the channel is filled with the same fluid as the gut surface. This allows phages to diffuse from the gut to the lymphatic and blood circulatory system. To obtain an upper bound limit to the number of phages penetrating the body by this mechanism, we neglect entropic effects associated to the section or number of channels, that is, we consider that multiple punctured points or a single hole with the same effective section lead to the same leaking. Under this assumption, phages will have the same diffusion constant both in the gut and in the channel. In the upper bound limit, we consider that phages diffuse as if they were in water at the body temperature (37 ⁰C). The diffusion constant, *D_w_*, as stated in the Einstein-Smoluchowski equation, is the ratio of the thermal energy, k_B_T, and the friction coefficient of phages in water, *𝛾_w_*, as given by Eq. (S4).

|  | 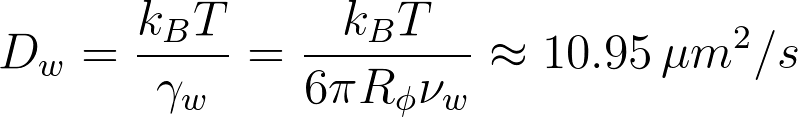 | Eq. (S4) |
| --- | --- | --- |

Here k_B_ is the Boltzmann constant. The friction constant is obtained by applying the Stokes-Einstein relation as shown in the denominator of the third term in Eq. (S4), where *R_𝜙_* is the effective radius of the phage; most phages are quasi-spherical and have a similar size to lambda phage, so we assume an effective radius of *R_𝜙_ ≈ 30 nm*. The viscosity of water at body temperature is *𝜈_w_ = 0.6913 mPa s I* (10). This leads to an approximate phage diffusion constant of *11 µm^2^/s*.

The flux of phages penetrating the body, *J_w_*, is proportional to the diffusion coefficient, *D_w_*, and the gradient of phage concentration in the transport channel as given by the Fick’s law:

|  | 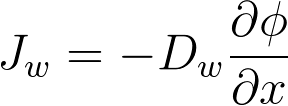 | Eq. (S5) |
| --- | --- | --- |

To calculate the flux, we need to determine the concentration profile of phages across the channel. The gradient of the flux is related to the change of phages per unit time by the continuity equation (conservation of mass), which leads to the diffusion equation:

|  | 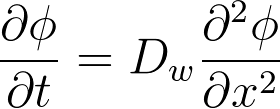 | Eq. (S6) |
| --- | --- | --- |

We consider that the side of the channel in the gut provides a constant supply of phages, *𝜙(0) = 𝜙_0_*, while in the other side of the channel (blood stream or lymphatic system) the phages do not accumulate, *𝜙(H) = 0,* where H is the “length” of the channel (height in Table 2). This will eventually lead to a stable concentration profile that does not change in time, i.e., it is stationary:

|  | 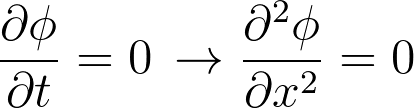 | Eq. (S7) |
| --- | --- | --- |

In this situation, the concentration of phages is determined by the Laplace equation—right term in Eq. (S7). Integrating this differential equation and applying the boundary conditions, *𝜙(0)* and *𝜙(H)*, give us the concentration profile:

|  | 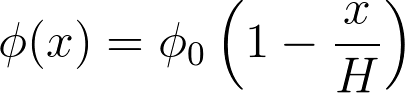 | Eq. (S8) |
| --- | --- | --- |

Applying this profile into the Fick’s law equation, Eq. (S4), we obtain the general expression of the flux of phages in the leaky-gut model:

|  | 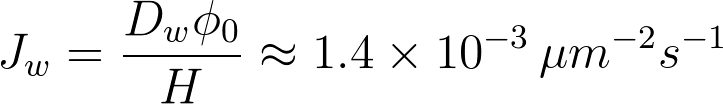 | Eq. (S9) |
| --- | --- | --- |

Applying the value of the phage diffusion in water at body temperature, *D_w_* (Eq. S3), the concentration of phage in the large intestine, *ϕ_0_ = ϕ_li_* (Table 2), and the height of the epithelial cell, H (Table 2), we obtain a flux of phages of 1.4 × 10^-3^ per unit area (µm^2^) and time (s).

How does this flux compare to the number of phages penetrating the body by the transcytosed mechanism? To answer this we estimate the effective section of the channel (or number of epithelial cells removed) necessary to lead to the same number of phages per day obtained in the gut in Eq. (S3). This condition is expressed as:

|  | 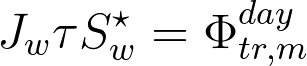 | Eq. (S10) |
| --- | --- | --- |

|  | 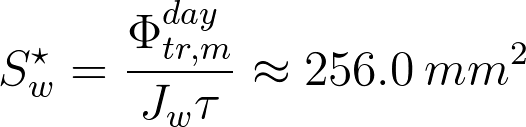 | Eq. (S11) |
| --- | --- | --- |

That is, the flux times the time (𝜏 = 24h) times the damaged surface (*S*_w_*) equates the number of phages transcytosed per day in the long intestine (Eq. S3). This leads to:

Taking into account the section of an epithelial cell, *S_ec_* (Table 2), we obtain that the number of damaged epithelial cells:

|  | 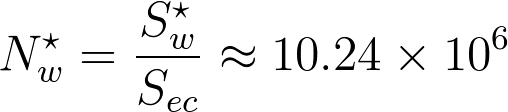 | Eq. (S11) |
| --- | --- | --- |

Thus, the leaky-gut mechanism requires more than ten million epithelial cells to be removed to reach a similar number of phages penetrating the body compared to the phage transcytosis mechanism. Notice that the flux in the leaky-gut model is an upper limit, so the number of damaged cells is a lower limit. If we introduce more realistic details in the model (e.g., wall effects, non-homogeneous flux across the section, entropic cost to enter the channel), this number could increase considerably.

**Table 1.** Experimental parameters of the T84 cell transcytosis assay.

| **Description** | **Parameter** | **Value** |
| --- | --- | --- |
| Volume of upper Transwell  Volume of lower Transwell  Surface area of a single Transwell  Time of incubation  Apical concentration of T4 phages applied to T84 cells  Basal concentration of T4 phages collected from T84 cells | *V_up_*  *V_lo_*  *S_lo_*  *t_in_*  *ϕ_up_*  *ϕ_lo_* | 250 𝜇l  250 𝜇l  1.12 cm^2^  2 h  4.70 × 10^7^ ml^-1^  6.99 × 10^3^ ml^-1^ |

**Table 2.** Physiological parameters associated with the large intestine.

| **Description** | **Parameter** | **Value** | **Reference** |
| --- | --- | --- | --- |
| Surface area (large intestine)  Volume (large intestine)  Number of epithelial cells  Dimensions of epithelial cells  Surface area (epithelial cell)  Number of phages in colon (in 0.41 litres of large intestine)  Concentration of phages in colon | *S_li_*  *V_li_*  *N_ec_*  width × length × height  *S_ec_*  *Φ_li_*  *ϕ_li_* | 3,460 cm^2^  409 ml  7.20 × 10^11^  5 𝜇m × 5𝜇m × 40 𝜇m  25 𝜇m^2^  2.09 × 10^12^  5.11 × 10^9^ ml^-1^ | Snyder *et al.,* 1975 ^1^  Sender *et al.,* 2016 ^2^  Snyder *et al.,* 1975 ^1^  Snyder *et al.,* 1975 ^1^  Snyder *et al.,* 1975 ^1^  Sender *et al.,* 2016 ^2^  *ϕ_li_= Φ_li_/V_li_* |

**Table 3.** Parameters associated with the transcytosed phages in humans model.

| **Description** | **Parameter** | **Value** | **Equation** |
| --- | --- | --- | --- |
| Phage transcytosis rate per unit time, unit area (wall), and phage apical concentration  Number of phage transcytosed per day in humans | *r_tr_*  *Φ^day^_tr_* | 0.166 μm/h  7.04 × 10^9^ phages/day | Eq. S1  Eq. S2 |
| Number of phage transcytosed per day in humans (mucus factor) | *Φ^day^_tr,m_* | 30.97 × 10^9^ phages/day | Eq. S3 |

**Table 4.** Parameters associated with the leaky gut model.

| **Description** | **Parameter** | **Value** | **Reference** |
| --- | --- | --- | --- |
| Boltzmann constant  Absolute temperature (body)  Phage effective radius  Water viscosity at body temperature | *k_B_*  *T*  *R_𝜙_*  𝜈_w_ | *1.38 x 10^-23^ K/J*  *310.15 K*  *30 nm*  *0.6913 mPa s* | –  *T = 273.15 +* t, where t = 37^o^C  Ackermann, 2007 ^3^  IAPWS, 2008 ^4^ |

**REFERENCES**

1. Snyder, W. S. *et al.* *Report on the Task Group on Reference Man*. (Pergamon Press: Oxford, 1975).

2. Sender, R. *et al.* Are We Really Vastly Outnumbered? Revisiting the Ratio of Bacterial to Host Cells in Humans. *Cell* **164,** 337–340 (2016).

3. Ackermann, H.-W. 5500 Phages examined in the electron microscope. *Arch. Virol.* **152,** 227–243 (2007).

4. Release on the IAPWS Formulation 2008 for the Viscosity of Ordinary Water Substance. (2008). Available at: http://www.iapws.org/.
