# Supplementary material for: Bacteriophage Transcytosis Provides a Mechanism To Cross Epithelial Cell Layers
Source: mBio. 2017 Nov 21;8(6):e01874-17. doi: 10.1128/mBio.01874-17 (PMC5698557; doi:10.1128/mBio.01874-17)
Supplement: TABLE S3 [file mbo006173601st3.pdf]

| Phage | Transcytosis percent | Capsid size (nm) | Tail size (nm) | Family | Bacterial host | Reference |
|---|---|---|---|---|---|---|
| T3 | 0.028% | 60 | 23 | Podoviridae | *Escherichia coli* | Fang *et al.,* [1] |
| T5 | 0.0157% | 90 | 250 | Siphoviridae | *Escherichia coli* | Effantin *et al.,* [2] |
| T7 | 0.0013% | 60 | 28 | Podoviridae | *Escherichia coli* | Cuervo *et al.,* [3] |
| SP01 | 0.005% | ~100 | ~160 | Myoviridae | *Bacillus subtilis* | Parker & |
| SPP1 | 0.0001% | 60 | 191 | Siphoviridae | *Bacillus subtilis* | Eiserling [4] |
| P22 | 0.0061% | 60 | 20 | Podoviridae | *Salmonella typhimurium* | Lhuillier *et al.,* [5]<br>Tang *et al.,* [6] |

## REFERENCES


1. Fang, P.-A. *et al.* Visualization of Bacteriophage T3 Capsids with DNA Incompletely Packaged In Vivo. *J. Mol. Biol.* **384,** 1384–1399 (2008).
2. Effantin, G., Boulanger, P., Neumann, E., Letellier, L. & Conway, J. F. Bacteriophage T5 Structure Reveals Similarities with HK97 and T4 Suggesting Evolutionary Relationships. *J. Mol. Biol.* **361,** 993–1002 (2006).
3. Cuervo, A. *et al.* Structural characterization of the bacteriophage T7 tail machinery. *J. Biol. Chem.* **288,** 26290–9 (2013).
4. Parker, M. L. & Eiserling, F. A. Bacteriophage SPO1 structure and morphogenesis. III. SPO1 proteins and synthesis. *J. Virol.* **46,** 260–9 (1983).
5. Lhuillier, S. *et al.* Structure of bacteriophage SPP1 head-to-tail connection reveals mechanism for viral DNA gating.
6. Tang, L. *et al.* Three-dimensional structure of the bacteriophage P22 tail machine. *EMBO J.* **24,** 2087–95 (2005).
