# Supplementary material for: Bacteriophage Transcytosis Provides a Mechanism To Cross Epithelial Cell Layers
Source: mBio. 2017 Nov 21;8(6):e01874-17. doi: 10.1128/mBio.01874-17 (PMC5698557; doi:10.1128/mBio.01874-17)
Supplement: TABLE S4 [file mbo006173601st4.pdf]

| Fraction | MDCK cells | | | A549 cells | | |
|---|---|---|---|---|---|---|
| | **Median ± s.d.** | ***n*** | **CV** | **Median ± s.d.** | ***n*** | **CV** |
| Applied | $2.7 \pm 3.1 \times 10^8$ | 6 | 82% | $2.1 \pm 2.8 \times 10^8$ | 5 | 77% |
| Cell Lysate | $2 \pm 1 \times 10^4$ | 5 | 53% | $2.6 \pm 2.3\times 10^4$ | 9 | 70% |
| 1 | 40 ± 41 | 4 | 87% | 30 ± 17 | 4 | 49% |
| 2 | 130 ± 149 | 4 | 104% | 100 ± 62 | 5 | 60% |
| 3 | 270 ± 193 | 3 | 84% | 80 ± 68 | 5 | 67% |
| 4 | 175 ± 284 | 4 | 99% | 155 ± 55 | 6 | 40% |
| 5 | 400 ± 194 | 3 | 66% | 310 ± 172 | 8 | 50% |
| 6 | $3.8 \pm 3.1 \times 10^3$ | 4 | 91% | $4.5 \pm 2.3 \times 10^3$ | 8 | 61% |
| 7 | 790 ± 304 | 5 | 38% | $2.9 \pm 0.7 \times 10^3$ | 8 | 24% |
| 8 | 755 ± 702 | 4 | 78% | $4.4 \pm 3.1 \times 10^3$ | 8 | 61% |
| 9 | 290 ± 55 | 3 | 21% | $3.1 \pm 1.4 \times 10^3$ | 8 | 43% |
| 10 | 10 ± 6 | 3 | 43% | 100 ± 66 | 8 | 46% |
