# Supplementary material for: Bacteriophage Transcytosis Provides a Mechanism To Cross Epithelial Cell Layers
Source: mBio. 2017 Nov 21;8(6):e01874-17. doi: 10.1128/mBio.01874-17 (PMC5698557; doi:10.1128/mBio.01874-17)
Supplement: TABLE S5 [file mbo006173601st5.pdf]

Solvent transcytosis			Inhibitors		Inhibitor transcytosis			One-way ANOVA *Non-parametric, Kruskal-Wallis*		One-way ANOVA *Parametric*	
**Median ± s.d.**	***n***	**CV**	**Inhibitor**	**Conc.**	**Median ± s.d.**	***n***	**CV**	**Mean rank diff.**	***P***	**Mean diff.**	***P***
$7.9 \pm 3 \times 10^4$	40	41%	Brefeldin A	5μg mL^{-1}	$2.3 \pm 2.2 \times 10^4$	8	73%	27.36	0.003	4.4×10^4	0.0004
$7.9 \pm 3 \times 10^4$	40	41%	Wortmannin	100 nM	$8.1 \pm 2.9 \times 10^4$	8	36%	-5.14	>0.99	-4.5×10^3	0.99
$7.9 \pm 3 \times 10^4$	40	41%	Bafilomycin	0.5 μM	$1 \pm .19 \times 10^5$	3	21%	-13.58	>0.99	-1.9×10^4	0.67
$7.9 \pm 3 \times 10^4$	40	41%	Chloroquine	100 μM	$5.3 \pm 2.7 \times 10^4$	4	42%	4.93	>0.99	1.1×10^4	0.90
$7.9 \pm 3 \times 10^4$	40	41%	W-7	100 μM	$8.2 \pm 1.1 \times 10^4$	8	14%	-6.95	>0.99	6.6×10^3	0.95
